# Clinical phenotypes of autism spectrum disorders and epilepsy comorbidity

**DOI:** 10.1192/j.eurpsy.2022.597

**Published:** 2022-09-01

**Authors:** T. Skrypnyk

**Affiliations:** SI «Research Institute of Psychiatry Ministry Of Health Of Ukraine», Department Of Mental Disoders Of Children And Adolescents, Kyiv, Ukraine

**Keywords:** autism spectrum disorders, epileptic seizures, repetitive behavior

## Abstract

**Introduction:**

ASD with epileptic seizures (ES) and/or specific epileptic activity on EEG (EEG SEA) and repetitive movements and vocalizations (RMV) can be determined by different variants of genetic polymorphism or by different variants of gene expression, determined by different influences.

**Objectives:**

To study the features of the clinical phenotype of ASD in preschool and school-age children with ES, EEG SEA and RMV.

**Methods:**

: The study group was divided 116 children aged 2-10 years with ASD into three subgroups: subgroup A - 23 children with a history of ES, subgroup B - 35 children with EEG SEA without ES, subgroup C - 19 children with ASD having EEG SEA, RMV (motor stereotypes, motor tics and/or vocal tics). The control group consisted of 39 children with ASD non a history of ES and EEG SEA.

**Results:**

: Children with ASD, complicated by severe and frequent ES are charactered by impaired social reciprocity and communication against the background of regression / stagnation of speech and motor skills development (aOR: 2,7 [1,7-4,8]). Disoders of communication and repetitive behavior in ASD in children of different ages are represented by different monoqualitative syndrome (phenotypes). Movement stereotypes predominated in children with ASD without ES and without EEG SEA (aOR: [1,8-7,9]). In children with ASD and EEG SEA, motor stereotypes and repetitive behavior were equally common ([SD] 2.1 -1, P =0.81).

**Conclusions:**

: Disorders of social reciprocity, communication, repetitive behavior are diagnostically significant for ASD complicated by ES, ASD with EEG SEA.

**Disclosure:**

No significant relationships.

